# The potency of HPLC-DAD and LC-MS/MS combined with ion chromatography for detection/purification of levulinic acid and bio-compounds from acid hydrolysis of OPEFB†

**DOI:** 10.1039/d2ra03563d

**Published:** 2022-10-07

**Authors:** Chatcha Saengsen, Orawan Sookbampen, Shuke Wu, Sasikarn Seetasang, Wichitpan Rongwong, Litavadee Chuaboon

**Affiliations:** Biomass and Oil Palm Center of Excellent, Walailak University Nakhon Si Thammarat 80160 Thailand; College of Life Science and Technology, Huazhong Agricultural University Wuhan 430070 China; National Nanotechnology Center (NANOTEC), National Science and Technology Development Agency Khlong Luang Pathum Thani 12120 Thailand; School of Engineering and Technology, Walailak University Nakhon Si Thammarat 80160 Thailand; School of Pharmacy, Walailak University Nakhon Si Thammarat 80160 Thailand litavadee.ch@wu.ac.th

## Abstract

This work reports a new strategy for the detection and purification of levulinic acid (LA) and bio-compounds from the acid hydrolysis and enzymatic treatment of oil palm empty fruit bunch (OPEFB) through high-performance liquid chromatography (HPLC) techniques combined with ion/ligand chromatography. The detections of LA, biomass-saccharides, hydroxymethylfurfural (HMF), and furfural were successfully elucidated by optimizing the multiple reaction monitoring mode (MRM) and liquid chromatography conditions using a Pb^2+^ ligand exchange column in the liquid chromatography with tandem mass spectrometry (LC-MS/MS) approach. High-performance liquid chromatography with diode-array detection (HPLC-DAD) combined with an H^+^ ion exchange column also showed potency for detecting chromophoric compounds such as LA, HMF, furfural, and acid (by-products) but not biomass-saccharides. Both techniques showed acceptable validation in terms of linearity, limit of detection (LOD), limit of quantitation (LOQ), accuracy, precision, and stability in both quantitative and qualitative analysis. However, the LC-MS/MS approach showed higher sensitivity for detecting LA and HMF compared with HPLC-DAD. Samples comprised of cellobiose, glucose, HMF, and LA from the acid hydrolysis of cellulose to LA with a mineral acid, and the biocatalysis of cellulase and β-glucosidase catalyzed cellulose (from OPEFB) to glucose were successfully monitored through the LC-MS/MS approach. In addition, using the optimal HPLC conditions obtained from LC-MS/MS, the purification of LA from other substances obtained from the hydrolysis reaction of cellulose (5 g) was successfully demonstrated by HPLC-DAD equipped with a fraction collector combined with an H^+^ ion exchange column at gram-scale of 1 g LA with a purification rate of 0.63 g ml^−1^ min^−1^.

## Abstract 2

The analytical approach for detection and purification levulinic acid from and bio-compound in hydrolysis biomass.

## Graphical abstract

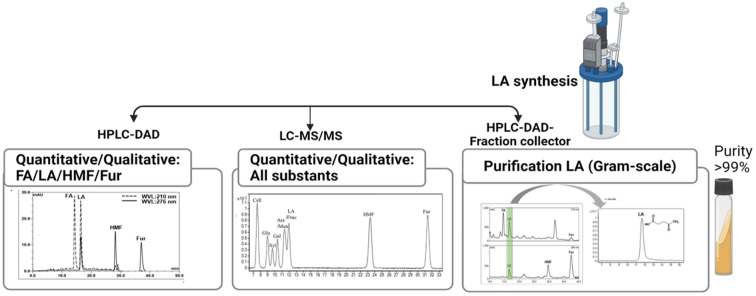

## 1. Introduction

Levulinic acid (LA) is an important biobased platform for synthesizing a variety of value-added chemicals.^1^ It can be used to produce liquid transportation fuels,^2,3^ food additives,^4^ herbicides,^5,6^ surfactants,^7^ and pharmaceutical compounds.^8^ Examples of LA derivatives are levulinate esters which can be used as fuel additives,^9^ 5-amino-levulinic acid, which is a herbicide,^5^ and γ-valerolactone which is a building block for polymer synthesis.^10^ Traditionally, LA is produced from fossil fuel resulting in an LA price of (20 $US per kg).^11^ Because of its high price and effects on the environment, the synthesis of LA has moved from using fossil fuels to renewable resources, such as lignocellulose biomass, which can significantly decrease the production price to only $US 2.5 per kg.^12^

**Fig. 1:**
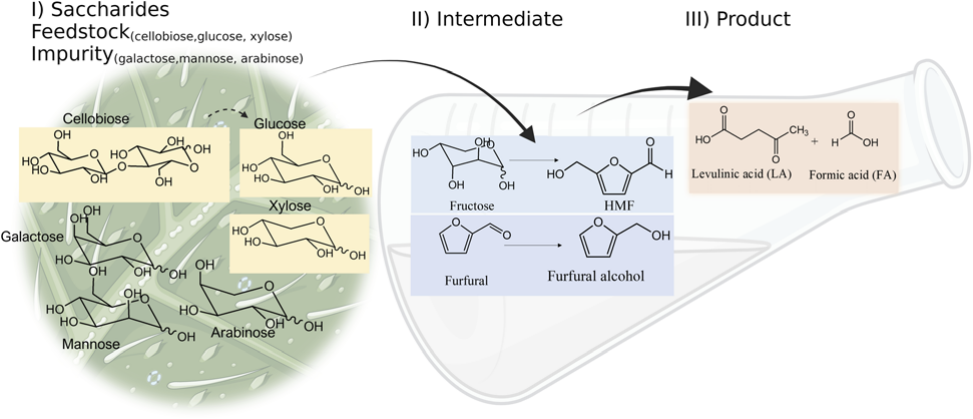
Pathways of LA synthesis through the C6-sugars (glucose) route and C5-sugars (xylose) route.

Oil palm is a major industrial crop in Southeast Asia.^13^ Its manufacturing and processing generate oil palm empty fruit bunch (OPEFB) as a by-product and waste. OPEFB is a rich lignocellulose source containing a high cellulose content of around 50% w/w and possesses the potential to generate LA up to 20–50% yield.^14,15^ LA can be synthesized from the lignocellulose biomass by a simple one-pot acid catalysis reaction using mineral acids or solid catalysts.^10,16–19^ Nevertheless, the one-pot LA reaction could result in other chemical compounds, such as hydroxymethylfurfural (HMF), furfural, and fructose, which are reaction intermediates, and acids which are considered as by-products (Fig. 1).^20^ In the acid catalysis reaction, the lignocellulose is firstly hydrolyzed to sugar substrates, such as glucose, xylose, and arabinose. Glucose is further decomposed to HMF, the intermediate in LA formation. As the reactions of the lignocellulose biomass to LA occur *via* isomerization, dehydration, and hydrolysis steps,^20^ it is essential to monitor the consumption of substrate sugar (derived from cellulose) and the alteration of the primary intermediates. This is key to developing an effective LA synthesis process and increasing the purity of the produced LA. The detection of intermediate compounds from the one-pot hydrolysis is also crucial for reducing the cost and time for LA production.^16,21^

High-performance liquid chromatography (HPLC) with ultraviolet-visible (UV-Vis), reflective index (RI), and evaporative light-scattering (ELS) detection is commonly employed to monitor the changes in the substrates, reaction intermediates, and products in LA synthesis.^22–26^ S. F. Chen *et al.* successfully monitored aliphatic acid, aromatic acid, aldehyde, and other phenolic degradation products with UV-Vis detection at 210 nm with a limit of quantitation (LOQ) ranging from 5 to 3000 ng ml^−1^.^22^ J. Liu *et al.* employed HPLC with UV-Vis detection at 210 nm and 286 nm to determine HMF, furfural, acetic acid, formic acid, and LA.^23^ The reported methods can determine the analytes with good analytical performance with linear regression coefficients of more than 0.999 and LOQ in the range of 5 to 3000 ng ml^−1^. Although the development of HPLC with a UV-Vis detector has shown great potential in the determination mentioned and listed in Table S1,† the detection is limited to chemical compounds containing chromophore groups. In the case of biomass-saccharides (substrate) and sugars (intermediates) without chromophore groups, a derivatization step is required, and the parameters in the derivatization reaction, such as time, pH, and temperature, need to be optimized.^27^ HPLC with RI detection is an alternative solution to determine the chromophore and non-chromophore chemicals. However, the RI detector lacks sensitivity.^26^ Its temperature dependence is unsuitable for the gradient elution of the mobile phase.^28^ The ELS detector appears to be more suitable for a gradient system in HPLC with better sensitivity compared to the RI detector,^29^ but it undesirably requires a combination of UV-Vis detectors or other detectors to detect chromophore compounds.^30–32^

Only limited types of HPLC columns have been tested for biomass-saccharides, HMF, furfural, and LA separation. C30-based columns were used. They were good at separating compounds such as LA, furfural, HMF, and acid. However, there was no report on sugar separation.^22^ Ion/ligand exchange columns such as H^+^, Na^+^, Ca^2+^, and Pb^2+^ have been investigated for biomass-saccharide separation because of their strong base anion exchange with the saccharide compounds.^33–35^ A study reported that the optimal separation of glucose and fructose in LA synthesis could be achieved using H^+^ ion-exchange columns.^36^ The stepwise separation of monosaccharides through the three Na^+^, Ca^2+^, Pb^2+^ ligand exchange columns were also appropriate for purifying monosaccharides. It was evident from the tests with marine particulate organic matter samples that the Pb^2+^ ligand exchange column showed potency for separating glucose, xylose, mannose, and galactose.^37^ Both H^+^ and Pb^2+^ columns showed high efficiency for the separation of saccharides.

Unfortunately, no study had successfully used a single HPLC approach to detect and purify LA, biomass-saccharides, intermediates (HMF, furfural), and by-products. Liquid chromatography-tandem mass spectrometry (LC-MS/MS) is a highly sensitive HPLC technique which can provide accurate methods for the targeted analysis of desired compounds. This technique has been used to detect biomass-derived compounds from hydrolysis synthesis. For example, Ning Shi *et al.* (2020) employed LC-MS/MS to analyze the hydrothermal conversion of HMF, furfural, and furfuryl alcohol, resulting in the detection of various carbocyclic compounds under hydrothermal conditions.^38^ Bevilaqua *et al.* (2013) applied LC-MS/MS to detect the hydrolysate composition of LA, 5-HMF, glucose, and xylose from the hydrolysis of rice husks.^39^ In these works, LC-MS/MS showed its potential to detect the substrates that occurred during the biomass one-pot hydrolysis reaction. However, there is no report of LC-MS/MS in the quantitative analysis of all compounds in LA production from biomass hydrolysis.

The current research focuses on the approach of LC-MS/MS in the detection of LA, intermediates, and by-products from biomass hydrolysis. We also compared LC-MS/MS validation in terms of the linearity, LOD (limit of detection), LOQ (limit of quantitation), accuracy, precision, and stability with HPLC-DAD (high-performance liquid chromatography with diode-array detection). Two HPLC columns of H^+^ and Pb^2+^ ion/ligand exchange were also employed to separate and purify LA from biomass hydrolysis. Actual samples of oil palm empty fruit bunch (OPEFB) were tested for the hydrolysis reaction. In addition, samples from the biocatalysis of OPEFB, a recent green technology used for biomass hydrolysis processes, were also tested. Finally, the conditions obtained from LC-MS/MS were employed with HPLC-DAD equipped with a fraction collector to purify LA in gram-scale production. Besides gaining a detection and purification approach, this study also explains how the ion exchange techniques separate the saccharides.

## 2. Materials and methods

### 2.1 Reagents

The standards, glucose, galactose, mannose, arabinose, xylose, fructose, cellobiose, HMF, furfural (Fur), levulinic acid (LA), and formic acid (FA) were purchased from Sigma-Aldrich (Darmstadt, Germany) and TCI (Japan) (>98% purity). Acetonitrile (ACN), ethanol, TFA and formic acid for HPLC and LC-MS grade were purchased from Honeywell Burdick & Jackson. The ultrapure water used in this work was produced by a Millipore MilliQ system. Pretreated OFPEB (high-purity cellulose >85%) was obtained from two-step treatment with peracetic acid (PA) and alkaline peroxide (AP).^40^

### 2.2 Stock solutions and bio-compounds from OPEFB hydrolysis reactions

The standard stock solutions were prepared and serially diluted in ultrapure water to their respective working solutions (mix of all analytes). Calibration curves in HPLC-DAD ranged from 1 to 15 mM (FA), from 1 to 20 mM (LA), and from 0.005 to 0.1 mM (HMF and Fur). Calibration curves in LC-MS/MS ranged from 0.07 to 1 mM (LA, Fur), from 0.005 to 0.1 mM (HMF), and from 0.01 to 10 mM (mono-di-saccharide). The mixed samples of LA and bio-compound were obtained from two methods, namely (1) from the hydrolysis reaction of oil palm empty fruit bunch (OPEFB) with a mineral acid, H_2_SO_4_ and (2) from the enzymatic hydrolysis of OPEFB. For the hydrolysis reaction of oil palm empty fruit bunch (OPEFB), 5 g of pretreated OPEFB (high-purity cellulose >85%) was placed in the acid catalyst (5% w/v H_2_SO_4_) under a high temperature of 170 °C for 1 h. Samples were collected at various time points and filtered by Microcon ultrafiltration before being analyzed by LC-MS/MS. For the enzymatic hydrolysis of OPEFB, a solution of 3.5 mg ml^−1^ of enzyme blend (cellulases, β-glucosidases, and hemicellulase) from Sigma-Aldrich, was mixed with 2.5 g of pretreated OPEFB (high-purity cellulose >85%) in 0.05 M sodium acetate buffer pH 5. The mixed solution in 10 ml was incubated at 50 °C, 150 rpm in an incubator shaker. Samples were collected at various time points and quenched by 60% acetonitrile. After removing denatured protein by centrifugation and Microcon ultrafiltration, a clear solution was analyzed using LC-MS/MS.

### 2.3 HPLC-DAD optimization

An HPLC with a DAD detector (Ulti-Mate 3000, Thermo Fisher Scientific) combined with a preparative H^+^ ion-exchange column (Hi-Plex H, 8 μm, 7.7 × 300 mm and Hi-Plex H guard column (8 μm, 7.7 × 50 mm) from Agilent Technologies) was used to detect FA, LA, HMF, and Fur. Optimization for compound separation was performed by considering the types of mobile phase, flow rate, and column temperature. 0.1% TFA, acetonitrile, and 5 mM H_2_SO_4_ were used as the mobile phase. The column temperature was optimized from 40 to 60 °C and the flow rate was adjusted from 0.6 to 1 ml min^−1^. Samples were analyzed in the isocratic elution mode with 20 μL injection volume. The wavelength in the DAD detector was tested at 210, 266, 276 and 286 nm to detect FA, LA, HMF, and Fur.

### 2.4 LC-MS/MS optimization

An Agilent 6490 triple quadrupole mass spectrometer with an electrospray ionization source (ESI) equipped with an ultra-performance liquid chromatography system (Agilent Technologies, USA) was used to detect cellobiose glucose, xylose, galactose, arabinose, mannose, LA, fructose, HMF, and Fur. The mass spectrometer detector conditions were set as follows: the capillary voltage was maintained at 4500 V, the nebulizer was set at 2 bars, the drying heater was set at 200 °C, and the drying gas flow was set at 8 L min^−1^. MRM mode (multiple reaction monitoring) was used to detect various standards. The standard samples were filtered through a Microcon ultrafiltration unit (10 kDa cutoff) and analyzed by LC-MS/MS to optimize the collision energy (CE). CE was varied in the 0–10 eV range on the triple quadrupole mass spectrometer. The highest abundance of product ions was selected for mass as MS2 in MRM mode. Then, the CE was varied again to select the suitable energy for the MRM condition of transition precursor ion : product ion ratio (MS1 : MS2). Data was analyzed by MassHunter software from Agilent Technologies. Fragmentation and dwell time were fixed at 380 V and 200 ms, respectively. The mode from optimization of MRM conditions in LC-MS/MS was set to MRM mode to monitor each standard and sample. MS/MS data collection for the standard plot was accomplished using quantitative analysis by the MassHunter software (Agilent). The preparative H^+^ ion exchange column and Pb^2+^ ligand exchange column (SP0810, 7 μm, 8 × 300 mm and SP-G guard column (10 μm, 6 × 50 mm) from Shodex) were compared for the separation of all compounds. The parameters of mobile phase and column temperature were optimized. Standards solutions were analyzed in both isocratic and gradient elution with 1 μL injection volume.

### 2.5 Evaluation of HPLC-DAD and LC-MS/MS performance

To evaluate the validation parameters for the HPLC-DAD method, different test concentrations of LA (1 to 20 mM), FA (1 to 15 mM), HMF, and Fur (0.005 to 0.1 mM) were prepared. To evaluate the validation parameters for the LC-MS/MS method, different test concentrations of cellobiose (0.018 to 1 mM), glucose (0.02 to 1 mM), xylose (0.1 to 10 mM), galactose (0.2 to 3 mM), arabinose (0.2 to 3 mM), mannose (0.2 to 2 mM), fructose (0.07 to 1 mM), LA (0.07 to 1 mM), HMF (0.005 to 0.1 mM), and Fur (0.07 to 1 mM) were prepared.

#### 2.5.1 Resolution (R_S_)

This was calculated with eqn (1):1
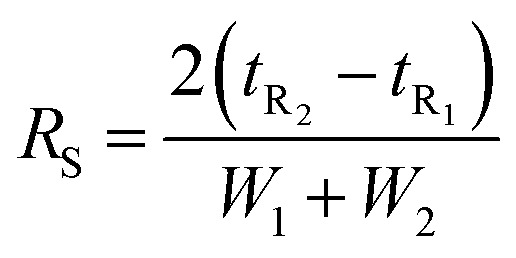
where *t*_R_ is the retention time of each peak, and *W* is the width of each peak.

#### 2.5.2 Linearity

The relationship between the acquired signal (peak area) and the analyzed concentration were then compared and expressed by linear regression. Acceptable linearity was obtained when the regression coefficient (*R*^2^) was higher than the criterion of 0.99.

#### 2.5.3 LOD (limit of detection) and LOQ (limit of quantitation)

The LOD and LOQ were calculated from the standard deviations of the slope's response and the calibration curve according to eqn (2) and (3):2
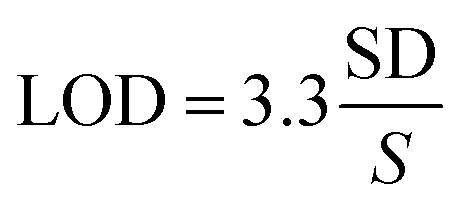
3
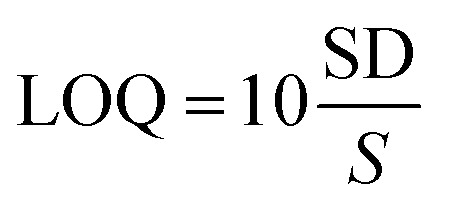
where SD is the standard deviation and *S* is the slope of the calibration curve.

#### 2.5.4 Accuracy

A mixed standard solution was employed for the accuracy tests at three concentrations and mixed with the real sample from acid-hydrolyzed OPEFB. The test was repeated three times; then the accuracy was calculated with eqn (4):4
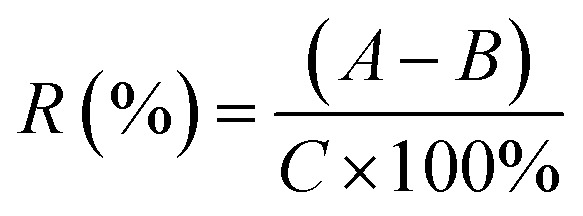
where *R* is the recovery, *A* is the measured concentration of the sample with the standard added, *B* is the original concentration of the analyte in the sample, and *C* is the theoretical concentration of the added standard. The contents of 4 analytes in each sample were determined.

#### 2.5.5 Precision

The method's precision was analyzed from two aspects: intra-day and inter-day. The mixed standard solution was injected six times a day for intra-day precision.

The same procedure was performed for three different days for inter-day precision. The relative standard deviation percentage (% RSD) of the peak areas of 4 analytes should be ≤2% to achieve precision. Calculation of the relative standard deviation was according to eqn (5):5
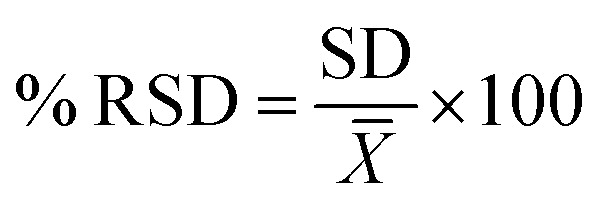
where RSD is the relative standard deviation, SD is the standard deviation and *X̄* is the average concentration.

#### 2.5.6 Stability

The stability was evaluated by analyzing the mixed standard solution at 0, 4, 8, 12, 24, and 48 h. By analyzing the peak area of each standard solution, the obtained % RSD was used to represent stability.

### 2.6 Gram-scale LA purification from the hydrolysis reactions through HPLC-DAD equipped with a fraction collector

The optimal methods obtained from HPLC-DAD and LC-MS/MS were employed in a system of HPLC-DAD equipped with a fraction collector. Samples from the hydrolysis reaction (200 μL) were injected into the system of HPLC-DAD equipped with a fraction collector. The purified LA was further identified through LC-MS/MS. Detailed information on the procedures for calculation of % yield and purification rate of LA are provided in ESI S4.†

## 3. Results and discussion

### 3.1 Development of HPLC-DAD procedure

The effects of HPLC parameters, including the types of mobile phase, temperature, and flow rates, were explored with the H^+^ ion-exchange column at the corresponding wavelength of 210 nm for FA detection and 266, 276, 284 nm for LA, HMF, and Fur detection, respectively. The resolution (*R*_S_) of the critical separated peaks of FA, LA, HMF, and Fur are shown in Table S2† (ESI).

An acceptable peak resolution (*R*_S_ > 1.5) was shown with the isocratic approach of both 0.1% TFA and 5 mM H_2_SO_4_ (Fig. S1†). It was found that the mobile phase of ACN/TFA could not separate FA and LA at a temperature of 50 °C although ACN has a high elution strength. However, 5 mM H_2_SO_4_ could not be employed with LC-MS/MS; therefore, 0.1% TFA was selected for the sake of further comparison. The comparison of column temperatures at 40 °C, 50 °C, and 60 °C showed that the highest temperature column at 60 °C could separate all the compounds in the lowest measurement time. Using a flow rate of 0.6 ml min^−1^ gave an unreasonably long measurement time. Therefore, stepwise flow rates were employed. FA and LA were well separated at a low flow rate of 0.6 ml min^−1^ in the first 25 min, while HMF and Fur were separated later using the second higher flow rate at 1 ml min^−1^ from 26 to 50 min.

**Fig. 2:**
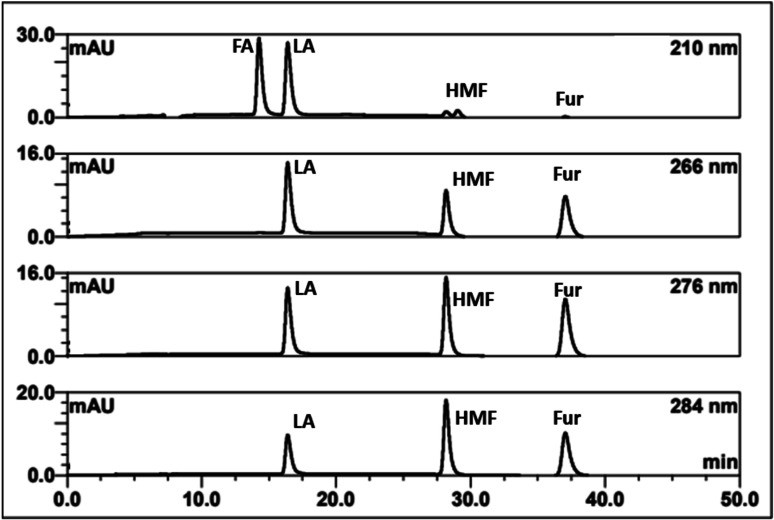
HPLC-DAD chromatograms of the separation of FA, LA, HMF, furfural (Fur) through an H^+^ ion exchange column with 0.1% TFA at 60 °C using detection at 210, 266, 276, and 284 nm.

In summary, the optimal condition with acceptable *R*_S_ value for HPLC-DAD were 0.1% TFA at 60 °C, and stepwise flow rates at 0.6 ml min^−1^ (0–25 min) and 1 ml min^−1^ (26–50 min). The chromatograms of the wavelengths at 266, 276, 284 nm, which focus on detecting LA, HMF, Fur, respectively, are shown in Fig. 2. Each of the wavelengths showed a similar acceptable validation (linearity of *R*^2^ > 0.99, accuracy of ∼100% recovery, and precision and stability of % RSD, relative standard deviation, ≤2%), as can be seen in Table 1. The highest *R*^2^ and % recovery and the lowest % RSD were obtained when the wavelength of 210 nm was selected for FA, and 276 nm was chosen to detect LA, HMF, and Fur.

**Table 1:** Validation results for HPLC-DAD methods in terms of the linearity, LOD, LOQ, accuracy, precision, and stability of the assay (*n* = 6) of the standard solution

Wave-length (nm)	Analytes	Linearity			LOD (mM)	LOQ (mM)	Accuracy	Precision		Stability
		Calibration curve	Range (mM)	*R* ^2^			% recovery	Inter-day % RSD	Intra-day % RSD	% RSD
210	FA	*y* = 1.1619*x* + 0.0308	1.000–15.000	0.9996	0.744	2.254	94.22	0.36	0.26	0.25
	LA	*y* = 0.0549*x* − 0.0119	1.000–20.000	0.9998	0.516	1.563	92.04	0.11	0.40	0.97
	HMF	*y* = 1.7049*x* + 0.1404	0.005–0.100	0.9975	0.010	0.031	55.20	1.68	1.83	1.78
266	LA	*y* = 0.0376*x* − 0.0112	1.000–20.000	0.9999	0.481	1.458	95.53	0.14	0.30	0.26
	HMF	*y* = 15.1400*x* − 0.1431	0.005–0.100	0.9960	0.013	0.039	106.61	0.28	0.30	0.33
	Furfural	*y* = 17.3910*x* − 0.0574	0.005–0.100	0.9850	0.025	0.075	87.82	0.56	0.29	1.18
276	LA	*y* = 0.0358*x* − 0.0121	1.000–20.000	0.9999	0.463	1.403	98.08	0.12	0.10	0.54
	HMF	*y* = 27.1070*x* − 0.1612	0.005–0.100	0.9989	0.008	0.023	84.35	0.09	0.12	0.16
	Furfural	*y* = 25.2890*x* − 0.2009	0.005–0.100	0.9995	0.004	0.014	92.20	0.44	0.39	1.07
284	LA	*y* = 0.0264*x* − 0.0153	1.000–20.000	0.9999	0.495	1.499	99.14	0.33	0.33	0.47
	HMF	*y* = 29.0090*x* + 0.2013	0.005–0.100	0.9977	0.025	0.077	118.22	0.07	0.22	0.19
	Furfural	*y* = 20.6860*x* − 0.0946	0.005–0.100	0.9996	0.036	0.013	89.55	0.38	0.26	1.99

### 3.2 LC-MS/MS procedure development

To explore the ability of LC-MS/MS in the separation/detection of sugars, apart from the mixture standards solutions of FA, LA, HMF and Fur, the saccharide and sugar compounds of cellobiose, glucose, xylose, galactose, arabinose, and mannose were also added into the testing solutions. Thus, the testing solution comprised 0.005–10 mM.

The conditions for the MRM (multiple reaction monitoring) were optimized to develop the separation method in LC-MS/MS. The fragmentation for each standard solution based on the signal of product ions in the difference CE is shown in Fig. S2.† The optimized collision energy (CE) in MRM mode is shown in Fig. S3.† Finally, the successful MRM conditions for detecting the ten-mixture standards solution are shown in Table 2.

**Table 2:** Optimized ESI-MS/MS parameters for the MRM determination of ten standards

Substances	Precursor ion (*m*/*z*)	Product ion (*m*/*z*)	Transition for MRM condition	Collision energy (eV)	Mode
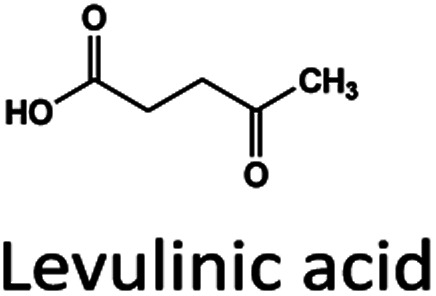	115	71, 99	115 : 71	7	Negative
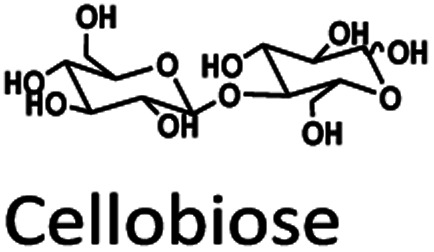	341	161, 179, 89	341 : 161	2	Negative
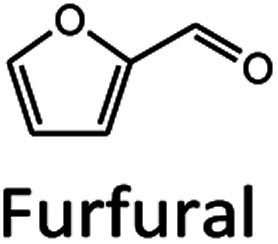	97	41,55	97 : 41	16	Positive
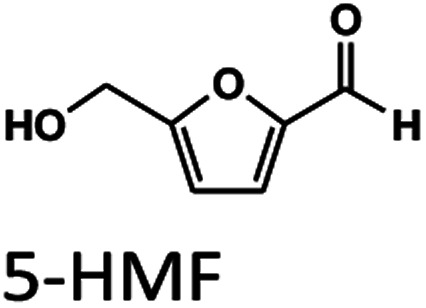	127	53, 81, 109	127 : 53	20	Positive
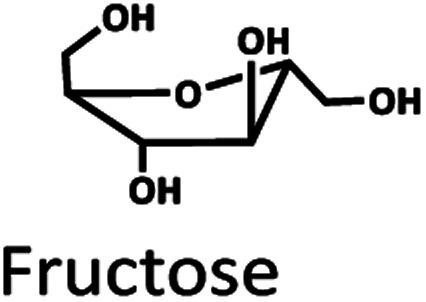	179	119, 89, 59	179 : 89	2	Negative
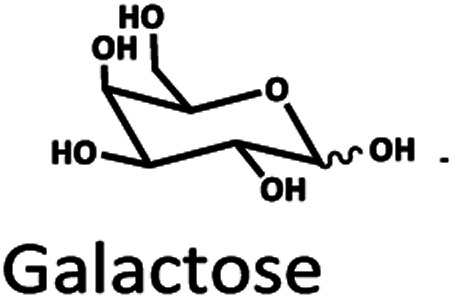	179	119, 89, 59	179 : 89	2	Negative
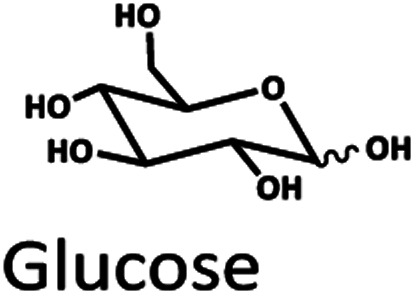	179	119, 89, 59	179 : 89	3	Negative
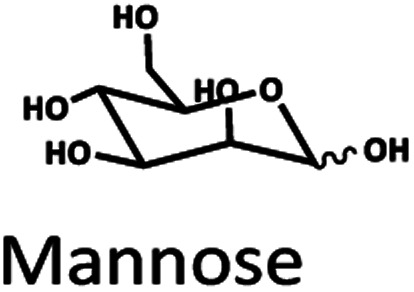	179	119, 89, 59	179 : 89	4	Negative
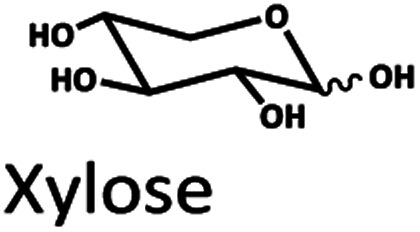	149	89, 59	149 : 89	1	Negative
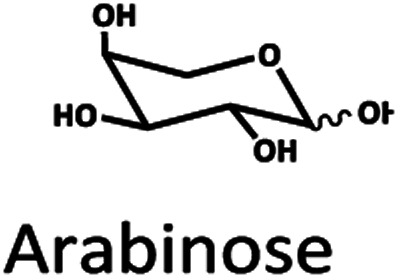	149	89, 59	149 : 89	1	Negative

**Fig. 3:**
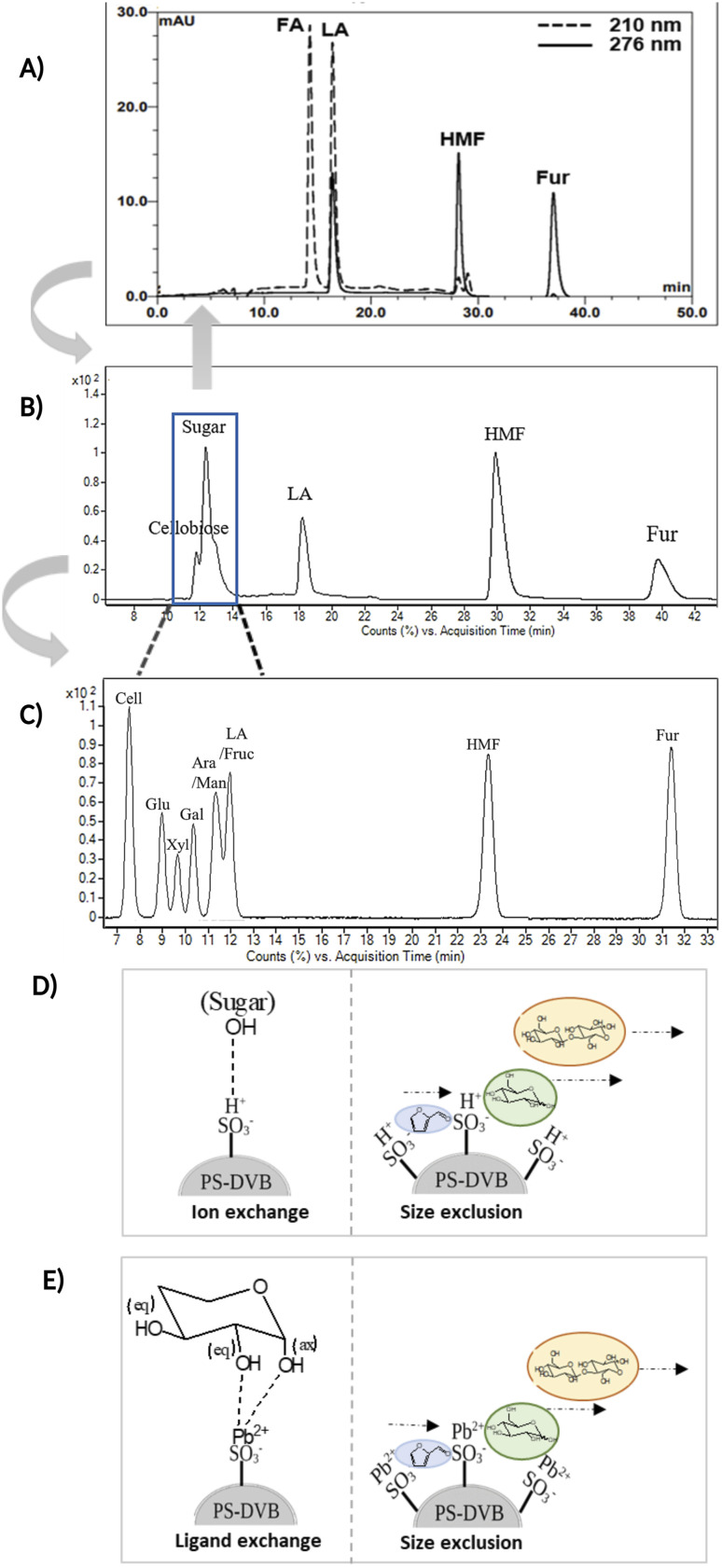
(A) HPLC-DAD chromatogram of the separation of FA, LA, HMF, and Fur in an H^+^ ion-exchange column with 0.1%TFA at 60 °C. (B) LC-MS/MS chromatogram of the separation LA, HMF, furfural (Fur), and various sugars in an H^+^ ion-exchange column with 0.1% TFA at 6 °C. (C) LC-MS/MS chromatogram of the separation LA, HMF, Fur and various sugars in a Pb^2+^ ligand exchange column with 0.1% FA at 80 °C. (D) The proposed ion exchange mechanism and size exclusion mode in the H^+^ column. (E) The proposed ligand exchange mechanism and size exclusion mode in the Pb^2+^ column.

Fig. 3A and B compare the chromatograms obtained from HPLC-DAD and LC-MS/MS using the H^+^ ion-exchange column. The optimal column conditions of 0.1% TFA at 60 °C, and the stepwise flow rates at 0.6 ml min^−1^ (0–25 min) and 1 ml min^−1^ (26–50 min) were employed in both devices. The LC-MS/MS chromatograms (Fig. 3B) showed the peaks of various sugars at 10–14 minutes, which were eluted from the column before LA. This suggests that the elution sequence through the H^+^ ion exchange column was sugars, LA, HMF, and Fur, respectively. However, it was found that the H^+^ ion-exchange column could not separate all the sugars, and only cellobiose was split from the group.

The Pb^2+^ ligand exchange column was then employed in LC-MS/MS, aiming to separate the sugars. Again, three of the HPLC column conditions, namely types of mobile phase, temperature, and flow rate, have been optimized in the Pb^2+^ column (see the results in Table S3 and Fig. S4†). The optimal conditions that could detect all the standards, including sugar compounds, were 0.1% FA at 80 °C with a flow rate of 1 ml min^−1^. The chromatogram using these condition is shown in Fig. 3C. Note that LA and fructose were eluted at the same retention at 12 min. We analyzed both compounds by extracting MRM mode in the different transitions (LA: 115 → 71; fructose: 179 → 89) to be able to distinguish both compounds.

The Pb^2+^ ligand exchange column showed better separation of most sugars than the H^+^ ion-exchange column (see Fig. 3B and C). We propose a mechanism following Goulding's study^41^ that the cation (Pb^2+^) can interact with pairs of –OH from sugars in axial (ax) and equatorial (eq) configurations (Fig. 3E). The Pb^2+^ ligand exchange column with high eight-coordinate geometry capability is more compatible with –OH in the ax/eq configuration of sugars (Fig. 3E) than the H^+^ ion-exchange column (Fig. 3D).^41,42^ Table S4 in ESI† shows the proposed number of pairs (ax–eq) from the interaction of –OH sugar with Pb^2+^. The elution sequence in each sugar depends on the number of pairs (ax–eq). Therefore, one pair (1p) in glucose and xylose was eluted first, and then three pairs (3p) of others followed.^41,43^ Since this column uses the size exclusion mode,^44^ the elution followed the sequence of disaccharides, monosaccharide C6, and monosaccharide C5, respectively.

**Fig. 4:**
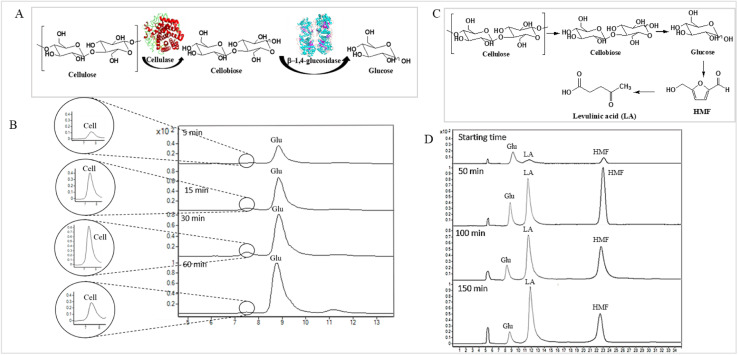
(A) Bioconversion of cellulose to glucose through cellulase and β-1,4-glucosidase. (B) Total ion chromatogram of LC MS/MS of the bioconversion of cellobiose (Cell) to glucose (Glu) through cellulase and β-1,4-glucosidase at various time points. (C) Acid hydrolysis reaction of converting cellulose to LA product. (D) Total ion chromatogram of LC MS/MS of the hydrolysis reaction of the conversion of cellulose from OPEFB to LA product at various time points.

The validation result of LC-MS/MS combined with the Pb^2+^ ligand exchange column is shown in Table 3. All analytes showed acceptable criteria for detection. However, the accuracy of the results for cellobiose, fructose, and impurity sugars obtained by comparison with actual hydrolysis compounds from the one-pot reaction could not be determined because no peaks were detected (Fig. 4D).

**Table 3:** LC-MS/MS linearity, LOD, LOQ, accuracy, precision, and stability of the assay (*n* = 6) of the standard solution^a^

Analytes	Linearity			LOD (mM)	LOQ (mM)	Accuracy	Precision		Stability
	Calibration curve	Range (mM)	*R* ^2^			% Recovery	Intra-day % RSD	Inter-day % RSD	% RSD
Cellobiose	*y* = 682 32*x* + 831.5700	0.018–1.000	0.9993	0.070	0.213	nd	1.55	0.41	1.29
Glucose	*y* = 693 76*x* + 316.5300	0.010–1.000	0.9994	0.070	0.213	82.43	1.84	0.34	1.64
Xylose	*y* = 8527.1*x* − 562.6000	0.100–10.000	0.9998	0.349	1.057	nd	0.58	0.41	1.25
Galactose	*y* = 1 021 30*x* + 21 545	0.200–3.000	0.9997	0.131	0.398	nd	1.82	0.55	1.28
Arabinose	*y* = 1068.9*x* + 299.3400	0.200–3.000	0.9912	0.671	2.032	nd	0.61	0.60	0.98
Mannose	*y* = 1 299 52*x* + 12 974	0.200–3.000	0.9991	0.153	0.463	nd	1.00	1.01	1.59
LA	*y* = 228 73*x* + 326.2900	0.070–1.000	0.9997	0.033	0.101	113.57	1.10	1.01	1.74
Fructose	*y* = 517 98*x* + 198.7700	0.070–1.000	0.9994	0.071	0.216	nd	1.87	0.38	1.16
HMF	*y* = 52 479 50*x* − 18.8750	0.005–1.000	0.9994	0.006	0.017	93.22	1.58	0.49	1.84
Furfural	*y* = 1 340 53*x* + 380.2600	0.070–1.000	0.9959	0.159	0.483	80.41	1.27	0.76	1.54

a nd = not detected.

To compare the validation of the analytical methodology of HPLC-DAD and LC-MS/MS, we employed the validation data in terms of the linearity, LOD, LOQ, accuracy, precision, and stability from Table 2 for HPLC-DAD (LA, HMF and Fur at 276 nm) and the data from Table 3 for LC-MS/MS. We used the validation data of the three compounds of LA, HMF and furfural to compare the validation of both techniques. The linearity of both techniques was over 0.995 in all three compounds. The LOD and LOQ of LA and HMF in the LC-MS/MS analysis were lower than those of HPLC-DAD. The LOD and LOQ were 13 times lower in the case of LA and 1.3 times lower in the case of HMF. This suggests that LC-MS/MS has higher sensitivity than HPLC-DAD for the detection of LA and HMF. However, the LOD and LOQ of furfural in HPLC-DAD showed greater sensitivity than LC-MS/MS, possibly due to the fragmentation parameter being fixed at 380 V by the limitation of our equipment. Other validations of accuracy, precision, and stability showed similar results for both techniques with an acceptable % RSD of less than 2. Note that FA cannot be detected in LC-MS/MS because the limit of the mass range in MRM mode is 50–1000 *m*/*z*.

### 3.3 Monitoring the consumption of substrate-sugar and intermediate-sugar for LA synthesis using enzymatic and acid hydrolysis reactions

The applicability of LC-MS/MS for detecting and monitoring the biocatalytic transformation of cellulose to glucose (Fig. 4A) and the acid hydrolysis of pretreated OPEFB to LA (Fig. 4C) was demonstrated. The results (Fig. 4B) indicate that LC-MS/MS could detect the decrease in cellobiose and increase in glucose from the enzymatic reaction of cellulase and glucosidase. In the acid hydrolysis, LC-MS/MS could also detect the change in glucose, LA, and HMF at various times (Fig. 4D). However, the C5 sugars route (mainly xylose, Fur) was not detected because high-purity cellulose was used as the substrate in the experiments.

### 3.4 Gram-scale purification of LA from hydrolysis reactions through HPLC-DAD equipped with a fraction collector

**Fig. 5:**
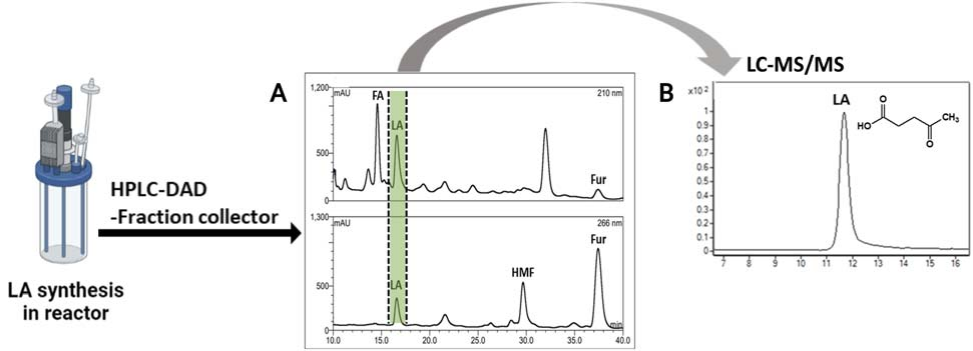
(A) Profile of LA chromatogram in HPLC-DAD equipped with fraction collector through an H^+^ ion preparative column. (B) Total ion chromatogram of purified LA (from fraction collector) by LC-MS/MS analysis.

The method verified from LC-MS/MS was employed with HPLC-DAD equipped with a fraction collector for LA purification at high purities. The injected samples were those of the solution from the acid hydrolysis of OPEFB. The result (Fig. 5A) showed a peak of LA in HPLC-DAD collected in the fraction collector. The collected solution showed a high purity of LA from LC-MS/MS analysis as only a peak of LA was showing (Fig. 5B). The potential of this method was shown by the successful purification of 1 g of LA (90.2 mM) with a purification rate of 0.63 g ml^−1^ min^−1^ and a 20% yield of LA (Table S5†). Industrial-scale LA purification methods have been investigated in various approaches, such as vacuum distillation, solvent extraction, steam stripping, membrane separation, adsorption, and ionic liquids.^45^ The steam stripping method showed the highest purity of LA among other methods of LA purification at industrial scales. Our result showed a higher purity of LA purification than the steam stripping method, which was in the range of 95–97% purity.^46^

## 4. Conclusion

Based on approaches for the separation and detection of LA, biomass-saccharides, and intermediates (HMF, furfural) from hydrolyzed biomass used in previous studies,^22–26^ we found they have limitations for the detection of saccharide compounds. This research fills the detection gap for both quantitative and qualitative analysis of LA, biomass-saccharides, and intermediate (HMF, furfural) through an LC-MS/MS technique. LC-MS/MS combined with a Pb^2+^ ligand exchange column successfully detected substrate-sugars and intermediate-sugars, HMF, furfural, and LA. However, HPLC-DAD still shows potency for detecting the chromophore compounds of LA, HMF, furfural (at 276 nm), and FA at 210 nm. A downstream method for the gram-scale purification of LA was also successfully implemented at the gram-scale using HPLC-DAD equipped with a fraction collector through an H^+^ ion preparative column. From these results it can be concluded that our new HPLC-DAD and LC-MS/MS strategy combined with ion/ligand exchange chromatography could be applied to purifying LA and detecting biomass-saccharides, LA, and intermediate (HMF, furfural) from the enzymatic and acid hydrolysis of OPEFB.

## Conflicts of interest

There are no conflicts to declare.

## Supplementary Material

RA-012-D2RA03563D-s001

RA-012-D2RA03563D-s002
