# Supplementary material for: The potency of HPLC-DAD and LC-MS/MS combined with ion chromatography for detection/purification of levulinic acid and bio-compounds from acid hydrolysis of OPEFB
Source: RSC Adv. 2022 Oct 7;12(44):28638–46. doi: 10.1039/d2ra03563d (PMC9539635; doi:10.1039/d2ra03563d)
Supplement: RA-012-D2RA03563D-s002 [file RA-012-D2RA03563D-s002.pdf]

Electronic Supplementary Material (ESI) for RSC Advances.
This journal is © The Royal Society of Chemistry 2022

Supporting Information

The potency of HPLC-DAD and LC-MS/MS combined with ion chromatography for purification/detection of levulinic acid and bio compound from OPEFB chemoenzymatic reactions

Chatcha Saengsen,[a] Orawan Sookbampen,[a] Shuke Wu,[b] Sasikarn Seetasang,[c] Wichitpan Rongwong, [a,d] and Litavadee Chuaboon*[a,e]

[a] Biomass and oil palm center of excellent, Walailak University, Nakhon Si Thammarat, 80160, Thailand

[b] College of Life Science and Technology, Huazhong Agricultural University, Wuhan, 430070, China

[c] National Nanotechnology Center (NANOTEC), National Science and Technology Development Agency, Khlong Luang, Pathum Thani 12120, Thailand

[d] School of Engineering and Technology, Walailak University, Nakhon Si Thammarat, 80160, Thailand

[e] School of Pharmacy, Walailak University, Nakhon Si Thammarat, 80160, Thailand

Correspondence should be addressed to Litavadee Chuaboon; litavadee.ch@wu.ac.th

S1. The previously studied of the separation and detection of LA pathway

Table S1. The reported studies of the separation and detection of LA pathway.

Method (Separation/detector)	Analyte	R^2	LOD/LOQ (µM)	Precision (RSD%)	Accuracy (%recovery)	Advantage/ Limitation
HPLC-UV (210nm)[1]<br><br>Column: C30	-FA<br>-HMF<br>-LA<br>-Furfural<br>-Acetic acid	0.9971<br>0.9997<br>0.9986<br>0.9836<br>0.9979	4.5/nd<br>0.088/nd<br>17/nd<br>0.267/nd<br>2.9/nd	4<br>1<br>13<br>1<br>1	104<br>104<br>103<br>101<br>101	**Advantage:**<br>Suitable for determination of aliphatic acid and aromatic acid in pretreatment biomass<br>**Limitation:**<br>No report of sugars detection
HPLC-RI [2]<br>Column: strong cation-exchange (H^+)	-HMF<br>-LA<br>-Furfural<br>-Acetic acid	>0.99	nd	nd	101.6% - 108.8%	**Advantage:**<br>Strong cation-exchange should be used for the HPLC analysis of biomass degradation compound<br>**Limitation:**<br>No report LOD/LOQ and sugars detection
HPLC-UV [3]<br>(286 nm: HMF, Fur<br>210 nm: FA, Acetic acid, LA)<br>Column: Mixed-Mode ion exchange and reversed-phase)	-FA<br>-HMF<br>-LA<br>-Furfural<br>-Acetic acid	0.9999<br>0.9999<br>0.9996<br>0.9993<br>0.9999	nd /32<br>nd /0.004<br>nd /26<br>nd /0.1<br>nd /16	0.78<br>0.69<br>0.95<br>0.86<br>0.48	103<br>101<br>100<br>98<br>99	**Advantage:**<br>Shot time in analysis from column mix mode<br><br>**Limitation:**<br>No report of sugars detection

nd= not detected

S2. HPLC -DAD

Table S2. Resolution (R_S) of HPLC-DAD condition with Hi-plex H (H+ ion exchange column).

Resolution values were calculated from equation in method S2.1. The wavelength at 210 nm was selected to calculate the resolution of FA, and 276 nm was chosen to calculate the resolution of LA, HMF, and furfural (Fur).

Figure	Mobile phase	Temperature (°C)	Flow rate (mL/min)	Resolution		
				$R_{S(LA\text{-}FA)}$	$R_{S(HMF\text{-}LA)}$	$R_{S(FUR\text{-}HMF)}$
a)	0.1%TFA: 20%ACN	50	0.6	1.17	3.09	3.62
b)	0.1%TFA	50	0.6	3.33	14.18	10.04
c)	5mM H_2SO_4	50	0.6	3.43	13.85	9.82
d)	0.1%TFA	40	0.6-1	4.24	16.56	10.60
e)	0.1%TFA	50	0.6-1	3.47	16.76	11.16
f)	0.1%TFA	60	0.6-1	2.95	16.03	10.08

Resolution values were calculated from equation in method S2.1.

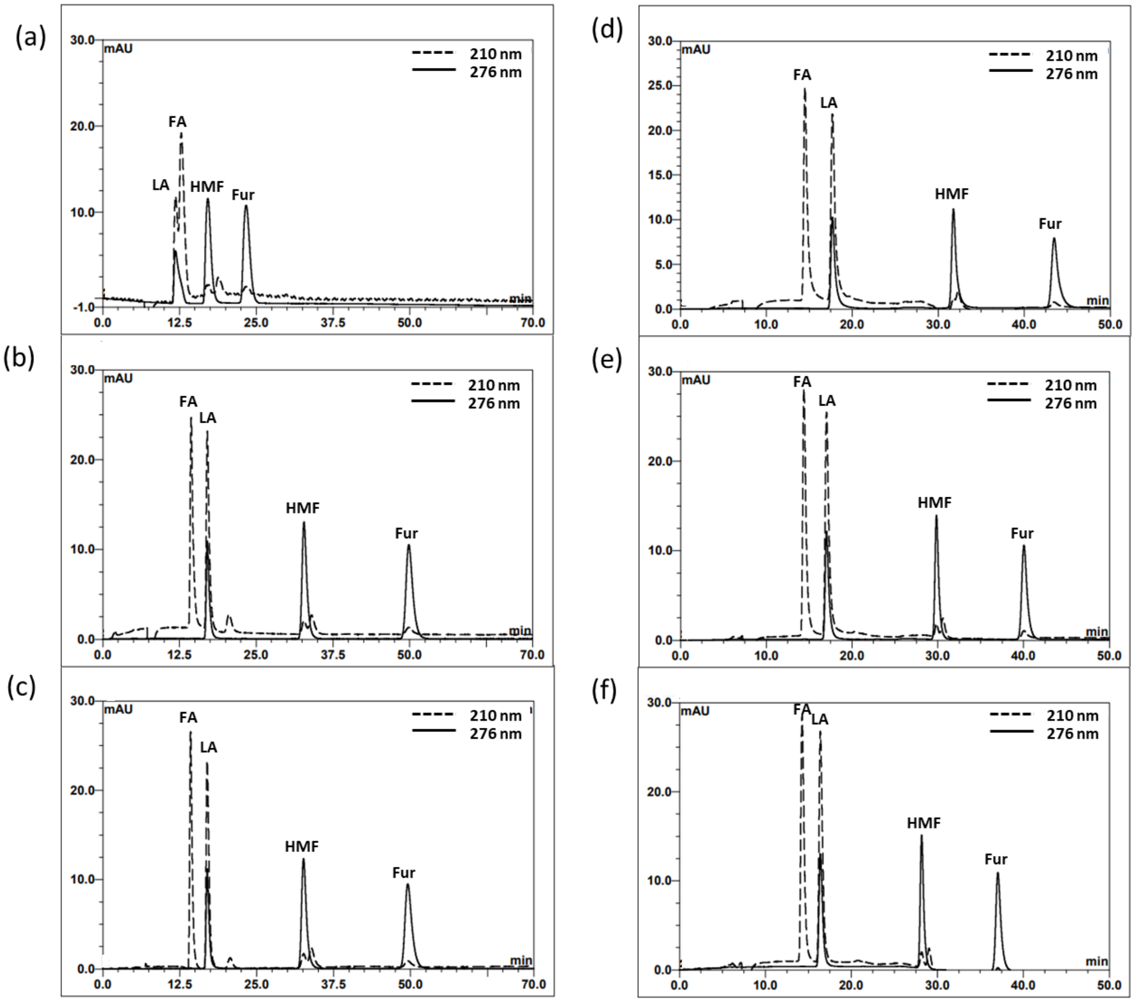

Fig. S1 HPLC–DAD chromatograms showing the separation of FA, LA, HMF, furfural (Fur) through H^+ ion exchange column in different HPLC conditions listed in table2. The separation condition at 50 °C in mobile phase of a) 0.1% TFA and 20% ACN, b) 0.1% TFA, and c) 5 mM H_2SO_4. The separation condition on mobile phase of 0.1% TFA at d) 40 °C, e) 50 °C, and f) 60 °C. The wavelength detection at 210 nm and 276 nm are illustrated as solid lines and dashed lines, respectively.

S3. Analytical parameters of each standards sample in MRM mode of LC-MS/MS

3.1 Product ion selection: Optimization MRM (Multiple Reaction Monitoring) conditions.
The highest abundant of product ion from this study was used as m/z of MS2 to generate transition MRM condition in each sugar.

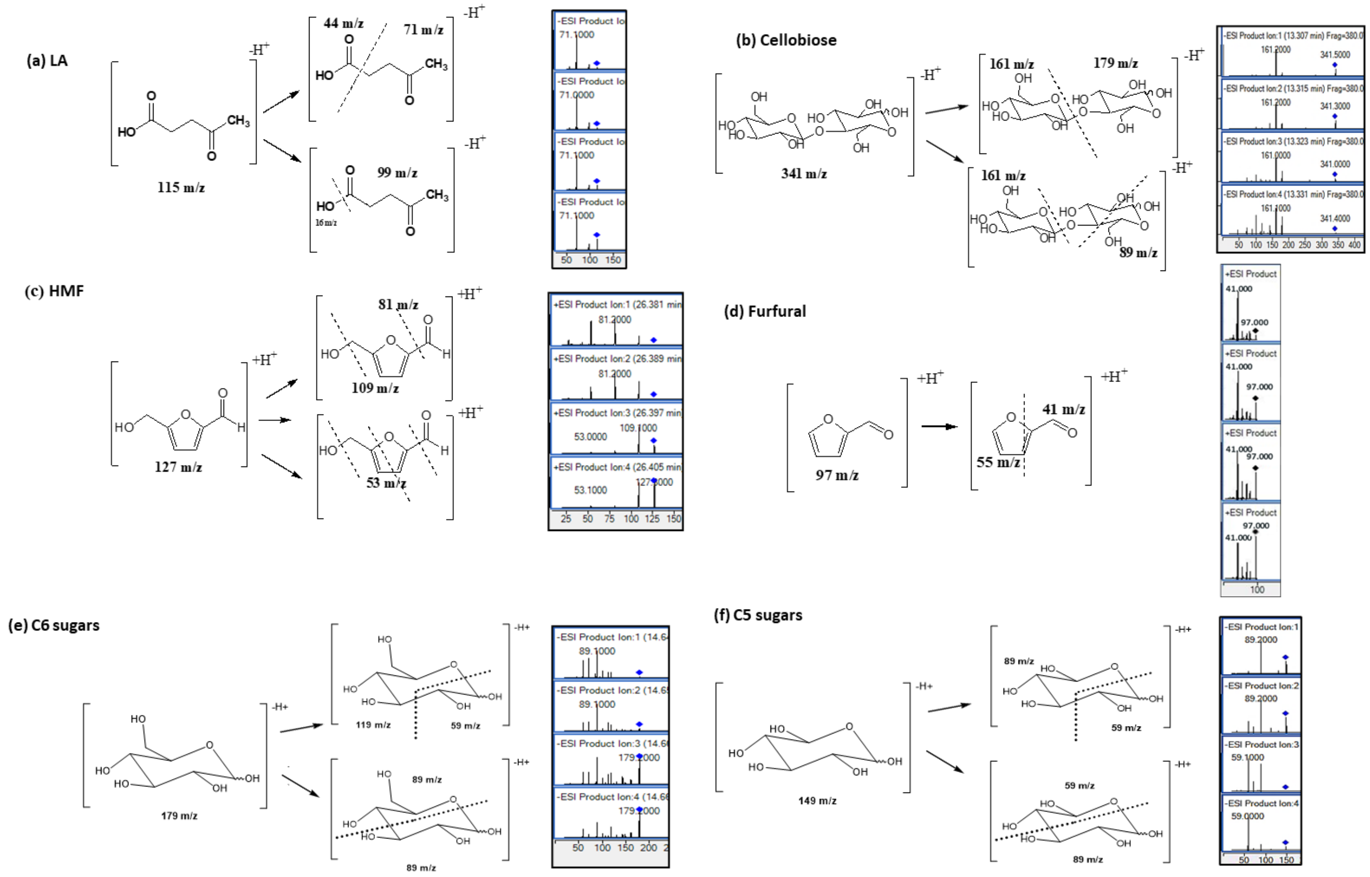

Fig. S2 Proposed fragmentation and product ion of LA, cellobiose, HMF, furfural, glucose (represent for C6 sugar), and xylose (represent for C5 sugar) were shown in (a), (b), (c), (d), (e), (f), respectively. The difference CE showed the different fragmentation patterns in each standard solution. C6 sugars as fructose, galactose, glucose, and mannose are shown the same fragmentation pattern of product ions at 119, 89, and 59 m/z (Figure S2E). C5 sugars as xylose and arabinose showed the same fragmentation pattern at 89 and 59 m/z (Figure S2F).

3.2 CE selection: Optimization MRM (Multiple Reaction Monitoring) condition.

In each transition MRM condition of various sugars, the collision energy was optimized to gain a high MS/MS analysis signal. The CE was varied in the range of 0-25 eV.

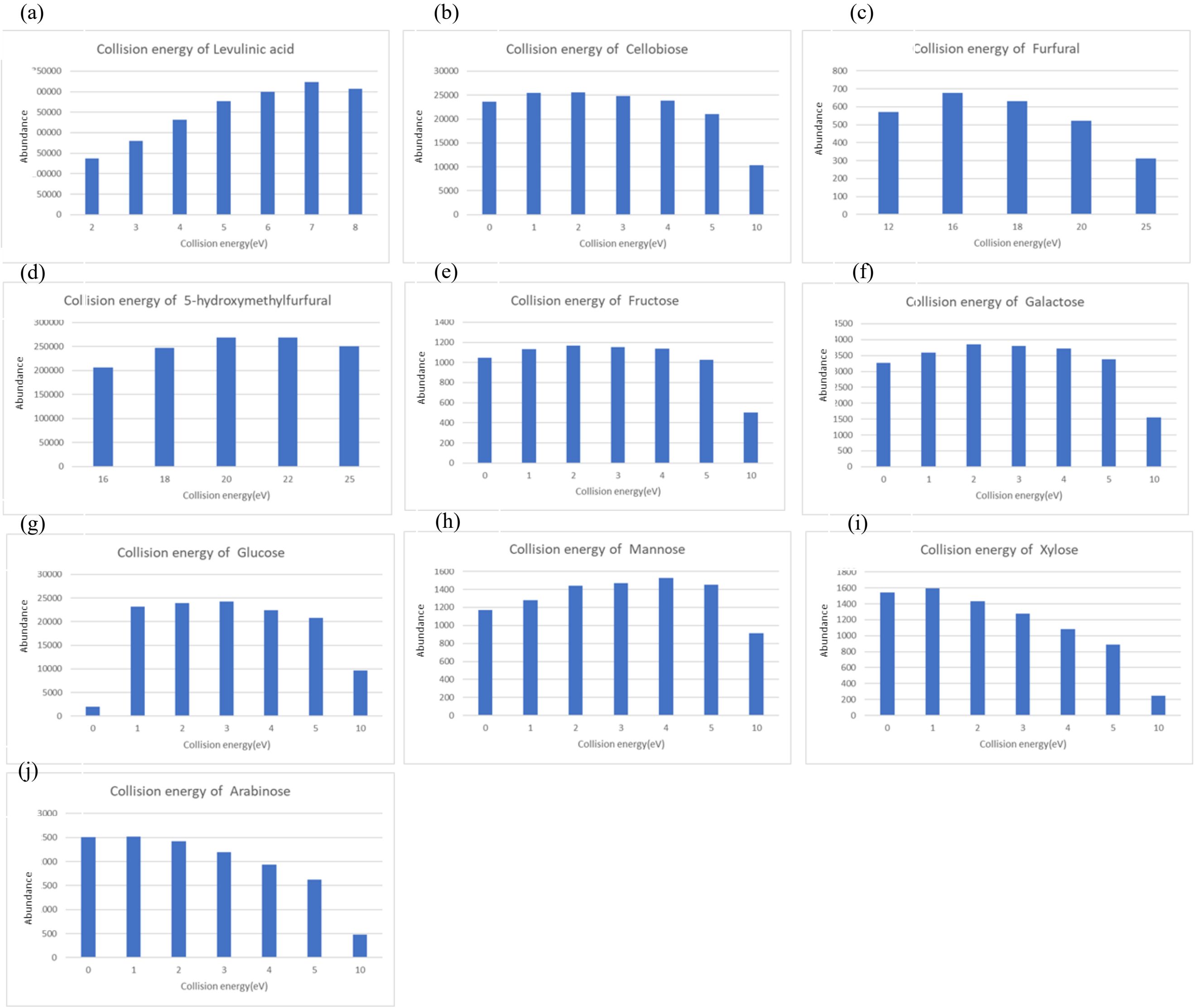

Fig. S3 The optimization CE in various standards of LA, cellobiose, furfural, HMF, fructose, galactose, glucose, mannose, xylose, and arabinose were varied for optimization the MRM condition and were showed in a, b, c, d, e, f, respectively.

Table S3. Resolution (R_S) of LC-MS/MS condition with SP0810 column (Pb^{2+} ligand exchange column).

Figure	Mobile phase	Tempera-ture (°C)	Flow rate (mL/min)	Resolution					
				$R_{s(Glu-Cell)}$	$R_{s(Xyl-Glu)}$	$R_{s\ (Gal-Xyl)}$	$R_{s(Ara-Gal)}$	$R_{s(Man-Ara)}$	$R_{s(Fruc-Man)}$
a)	0.1%FA+5%ACN	80	0.6	1.59*	1.11	1.28	0.75	0.31	0.60
b)	0.1%FA+20%ACN	80	0.6	1.57*	0.28	1.56*	0.63	0.64	0.76
c)	0.1%FA+5%ACN	65	1	1.53*	0.76	0.90	0.98	0.24	0.84
d)	0.1%FA+20%ACN	65	1	1.56*	0.29	1.82*	0.86	0.65	1.13
e)	0.1%FA	80	1	1.72*	0.97	0.95	1.19*	0.19	0.66

* Acceptable Resolution (R_S) >1.5

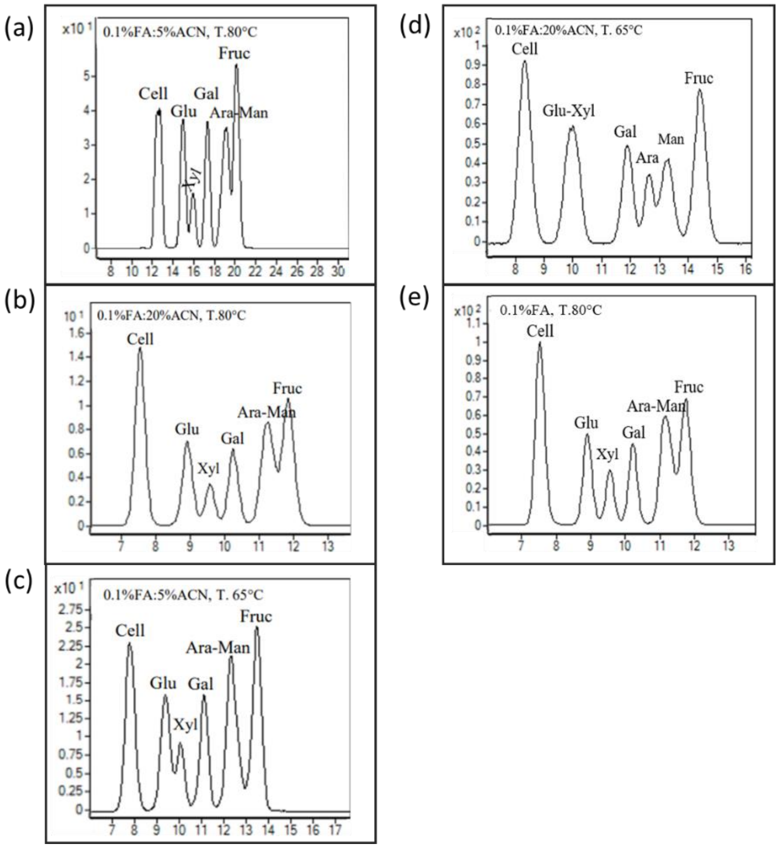

Fig. S4 LC-MS/MS chromatogram of separation cellobiose (Cell), glucose (Glu), xylose (Xyl), galactose (Gal), arabinose (Ara), mannose (Man), and fructose (Fruc) through Pb^{2+} ligand exchange column in different HPLC conditions following table S3.

Table S4. **Proposed effect of combination of ligand exchange and size exclusion modes in Pb^{2+} column**

Sugars	Retention time (min)	Effect of size exclusion (SEC)	Effect of Ligand exchange (Propose number of the pair from interaction of -OH sugar, with Pb^{2+})
Cellobiose	7.546	Disaccharide	1p
Glucose	8.904	Monosaccharide C6	1p (1p from α-anomer, low amount in β-anomer)
Xylose	9.574	Monosaccharide C5	1p (1p from α-anomer, low amount in β-anomer)
Galactose	10.231	Monosaccharide C6	3p (2p from α-anomer, 1p from β-anomer)
Arabinose	11.098	Monosaccharide C5	3p (1p from α-anomer, 2p from β-anomer)
Mannose	11.294	Monosaccharide C6	3p (1p from α-anomer, 2p from β-anomer)
Fructose	11.763	Monosaccharide C6	3p (2p from α-anomer, 1p from β-anomer)

p= pair (ax-eq).

S4. Semi-large scale purification LA from hydrolysis reactions through HPLC-DAD equipped with fraction collector

S4.1 %Yield of LA was calculated by equation (6):

$$\%\ \text{yield} = \frac{C \times V_R}{m_S} \times 100 \ldots\ldots (6)$$

where C is the concentration of product (g/ ml), V_R is the volume of reactant (mL), and m_S is the mass of substrate (g).

S4.2 Rate of purification (g/mL/min) was calculated by equation (7):

$$\text{Rate purification} = \frac{\text{mass of product(g)}}{\text{flow rate of HLPC(mL/min)}} \ldots\ldots (7)$$

Table S5. The purification LA from the hydrolysis OPEFB through HPLC-DAD equipped with fraction collector through H+ ion preparative column.

Analysts	%Yield	Rate of purification (g/ml/min)
FA	4.99	0.15
LA	20.95	0.63
HMF	0.02	0.00075
FUR	0.20	0.01

Reference

1. S.-F. Chen, R. A. Mowery, V. A. Castleberry, G. P. v. Walsum and C. K. Chambliss, *Journal of Chromatography A*, 2006, **1104**, 54-61.
2. R. Xie, M. Tu, Y. Wu and S. Adhikari, *Bioresource Technology*, 2011, **102**, 4938-4942.
3. J. Liu, J. Li, J. Zheng and C. Wang, *Carbohydrate Polymers*, 2017, **173**, 150-156.
